# A novel pancreatic cancer model originated from transformation of acinar cells in adult tree shrew, a primate-like animal

**DOI:** 10.1242/dmm.038703

**Published:** 2019-04-15

**Authors:** Qiu Tu, Dong Yang, Xianning Zhang, Xintong Jia, Sanqi An, Lanzhen Yan, Hongjuan Dai, Yuhua Ma, Chengwei Tang, Weimin Tong, Zongliu Hou, Longbao Lv, Jing Tan, Xudong Zhao

**Affiliations:** 1 Key Laboratory of Tumor Immunological Prevention and Treatment of Yunnan Province, Central Laboratory of Yan'an Hospital, Affiliated to Kunming Medical University, Kunming, Yunnan 650051, China; 2 Key Laboratory of Animal Models and Human Disease Mechanisms of Chinese Academy of Sciences/Key Laboratory of Bioactive Peptides of Yunnan Province, Kunming Institute of Zoology, Kunming, Yunnan 650223, China; 3 Department of Gastroenterology, West China Hospital, Sichuan University, Chengdu 610041, China; 4 Kunming Primate Research Center, Chinese Academy of Sciences, Kunming, Yunnan 650223, China; 5 Department of Pathology, Institute of Basic Medical Sciences and Neuroscience Center, Chinese Academy of Medical Sciences and Peking Union Medical College, Beijing 100005, China; 6 Center for Excellence in Animal Evolution and Genetics, Chinese Academy of Sciences, Kunming, Yunnan 650223, China

**Keywords:** Pancreatic cancer, Animal model, Tree shrew, Acinar cells, Cdkn2b

## Abstract

Pancreatic cancer is one of the most lethal common cancers. The cell of origin of pancreatic ductal adenocarcinoma (PDAC) has been controversial, and recent evidence suggested acinar cells as the most probable candidate. However, the genetic alterations driving the transformation of pancreatic acinar cells in fully mature animals remain to be deciphered. In this study, lentivirus was used as a tool to introduce genetic engineering in tree shrew pancreatic acinar cells to explore the driver mutation essential for malignant transformation, establishing a novel tree shrew PDAC model, because we found that lentivirus could selectively infect acinar cells in tree shrew pancreas. Combination of oncogenic *KRAS^G12D^* expression and inactivation of tumor suppressor genes *Tp53*, *Cdkn2a* and *Cdkn2b* could induce pancreatic cancer with full penetrance. Silencing of *Cdkn2b* is indispensable for Rb1 phosphorylation and tumor induction. Tree shrew PDAC possesses the main histological and molecular features of human PDAC. The gene expression profile of tree shrew PDAC was more similar to human disease than a mouse model. In conclusion, we established a novel pancreatic cancer model in tree shrew and identified driver mutations indispensable for PDAC induction from acinar cells in mature adults, demonstrating the essential roles of Cdkn2b in the induction of PDAC originating from adult acinar cells. Tree shrew could thus provide a better choice than mouse for a PDAC model derived from acinar cells in fully mature animals.

## Abstract (precis)

**Summary:** Our work identified the driver mutations indispensable for PDAC induction from acinar cells in mature adults and established a novel PDAC animal model with increased similarity to human disease.

## INTRODUCTION

Pancreatic cancer is the fourth leading cause of cancer-related deaths in the USA and was projected to be the second leading cause by 2030 (Rahib et al., 2014). Pancreatic ductal adenocarcinoma (PDAC) accounts for 90% of pancreatic cancer. The pancreas is mainly composed of acinar, ductal and islet cells. Although all are proposed as potential candidates for the cell of origin for PDAC, increasing evidence suggests that the acinar cells are the most likely candidate (Cubilla and Fitzgerald, 1976; Furukawa et al., 1994; Kopp et al., 2012; Kozuka et al., 1979; Maresch et al., 2016; Pour et al., 1997).

The expression of oncogenic KRAS^G12D^ at embryonic stages can induce pancreatic cancer in mice, which is accelerated by inactivation of *Trp53*, *Cdkn2a* or *Smad4*, genes that are frequently altered in human PDAC. However, expressing KRAS^G12D^ in adult (postnatal day >60) mouse acinar cells could not induce visible lesions, even in combination with knockout of tumor suppressor *Trp53* or *Cdkn2a* (Guerra et al., 2011), which suggests the importance of generating PDAC models via transformation of adult cells *in vivo*.

The tree shrew (*Tupaia belangeri chinensis*) is a primate-like animal that possesses a closer genetic relationship to humans than rodents (Fan et al., 2013; Xiao et al., 2017). It has been proposed as a viable animal model alternative to primates for biomedical research and drug-safety testing (Cao et al., 2003; Yan et al., 2016; Yao, 2017), and used to model human diseases, including cancers such as hepatocellular carcinoma (Park et al., 2000; Su et al., 2003), breast cancer (Ge et al., 2016; Xia et al., 2012) and glioblastoma (Tong et al., 2017).

In this study, we found that lentivirus could specifically infect acinar cells in adult tree shrew. By taking advantage of this, a tree shrew model of PDAC derived from acinar cells was established by overexpressing mutant KRAS^G12D^ and inactivating the tumor suppressor genes *Tp53*, *Cdkn2a* and *Cdkn2b*. *Cdkn2b* inactivation was required for PDAC formation instead of a passenger deletion due to juxtaposition of *Cdkn2a*. Histological and immunohistochemical examinations revealed that the pancreatic cancer was similar to moderately differentiated PDAC in humans. Moreover, the gene expression profile of tree shrew PDAC was more similar to that of human PDAC than the mouse model.

## RESULTS

### Selective lentiviral infection of pancreatic acinar cells in tree shrew

**Fig. 1.**
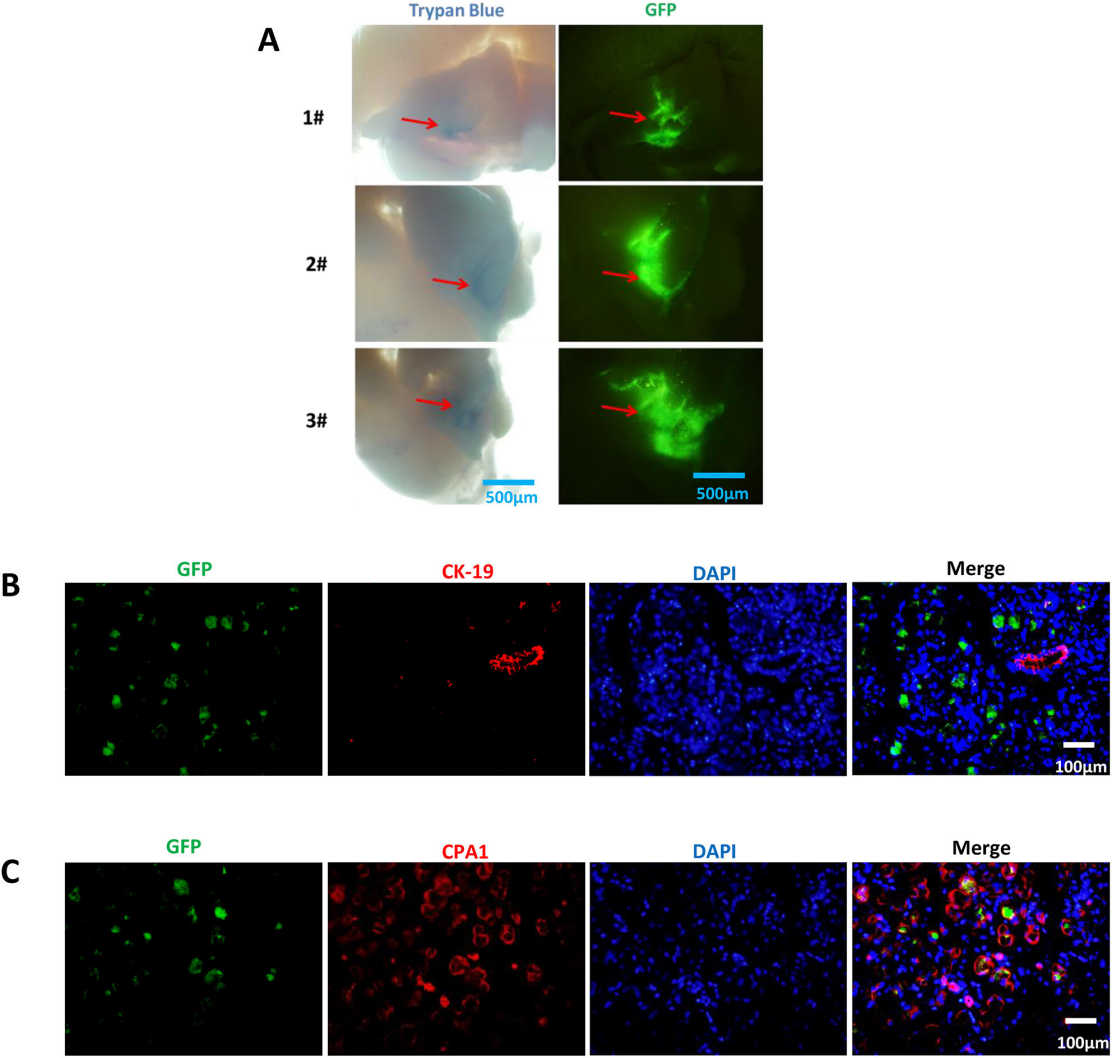
**Lentivirus specifically infects acinar cells in tree shrew pancreas.** (A) Three animals were injected with EGFP-expressing lentivirus; green fluorescence was observed on day 4 around the injection site (red arrows). (B,C) Double labeling for GFP (green) and CK19 (red) (B) and GFP (green) and CPA1 (red) (C) in acinar cells. 4′,6-diamidino-2-phenylindole (DAPI; blue) was used to stain nuclei.

To determine whether pancreatic tissue can be infected by lentivirus in tree shrew, and to establish which types of pancreatic cells are infected, the pTomo-EGFP lentivirus was injected into tree shrew pancreas and EGFP expression was examined 4 days later. EGFP was highly expressed around the injection site (Fig. 1A). Immunohistochemical detection of EGFP and the acinar cell-specific marker carboxypeptidase A1 (CPA1) or ductal cell marker cytokeratin 19 (CK19) showed that all EGFP-positive cells were CPA1 positive, whereas no EGFP/CK19 double-positive cells were detected (Fig. 1B,C). These results suggest that the lentivirus could infect pancreatic acinar cells in adult tree shrew.

### Induction of pancreatic cancer in adult tree shrew

**Fig. 2.**
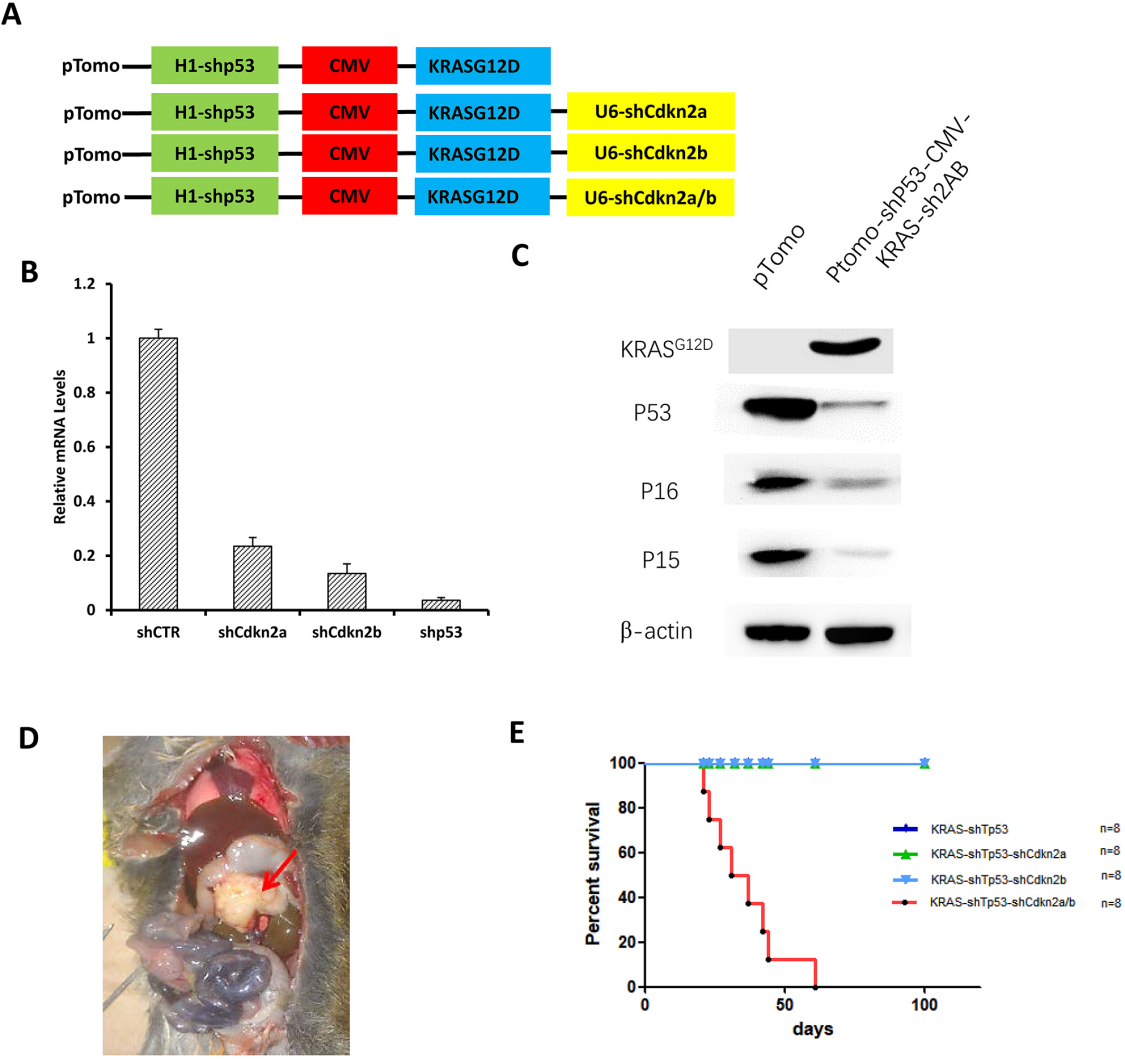
**Induction of pancreatic cancer in tree shrew.** (A) Schematic illustration of lentiviral vectors. (B) shRNA-mediated knockdown efficiency in a tree shrew liver cell line. (C) Western blot analysis of KRAS^G12D^, P53, P16 and P15 expression in tree shrew liver cells 4 days after lentiviral infection. (D) A tumor mass was observed at the head of the pancreas (red arrow). (E) Survival curve of the tree shrew pancreatic cancer model. The mortality of the KRAS-shTp53-shCdkn2a/b group was 100%, but there were no tumors or deaths recorded in animals of the KRAS-shTp53, KRAS-shTp53-shCdkn2a and KRAS-shTp53-shCdkn2b groups.

To explore the driver mutations of pancreatic cancer from malignant transformation of acinar cells in adult tree shrew, several lentiviral vectors were constructed to express KRAS^G12D^ and silence *Tp53*, *Cdkn2a*, *Cdkn2b* or *Cdkn2a/b* (Fig. 2A). Expression of KRAS^G12D^ and knockdown efficiency of short hairpin RNAs (shRNAs) targeting *Tp53*, *Cdkn2a* and *Cdkn2b* were evaluated at mRNA and protein levels in a tree shrew liver cell line (Tong et al., 2017). Lentiviral infection resulted in efficient expression of KRAS^G12D^ and inactivation of P53, P16 and P15 (Fig. 2B,C).

The sexual maturation time of tree shrew is 4-6 months and life span is 6-8 years. Two- to three-year-old adult tree shrews were used to ensure full maturity. Orthotopic injection of 1×10^7^ KRAS-shTp53-shCdkn2a/b lentiviral particles into the head of the pancreas induced tumor formation with full penetrance after 3-7 weeks (Fig. 2D,E). The animals exhibited other gross morphological changes associated with pancreatic cancer, such as intestinal darkening, colorectal inflation, gall bladder dilatation and blockage of bile overflow (Fig. 2D). In contrast, there was no tumor formation in animals injected with other lentiviral particles. These results suggest that expression of oncogenic *Kras* along with loss of function of *Tp53*, *Cdkn2a* and *Cdkn2b* were indispensable for transformation of adult acinar cells and induction of pancreatic cancer.

### Characterization of pancreatic cancer in tree shrew

**Fig. 3.**
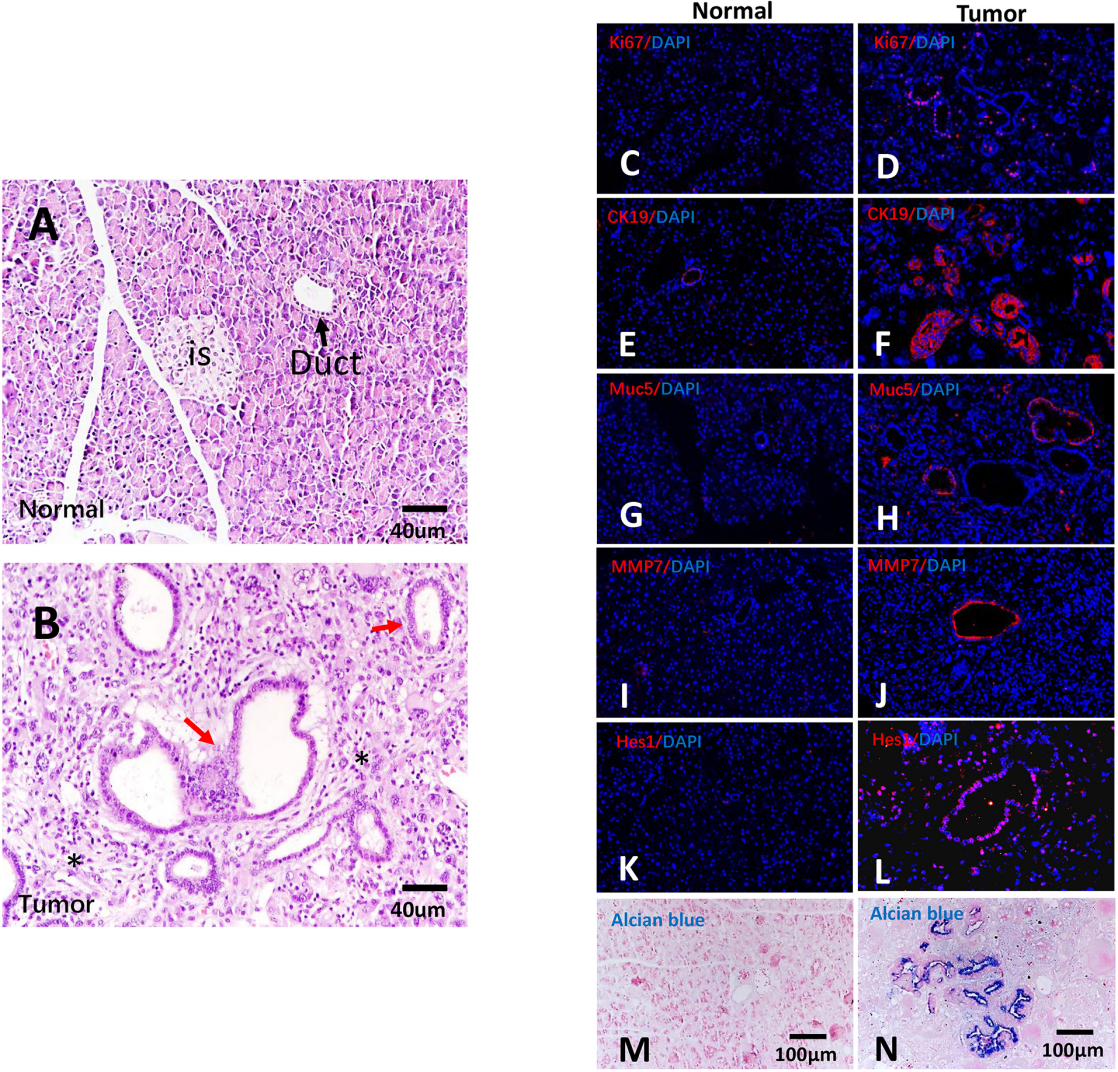
**Characterization of pancreatic cancer in tree shrew.** (A,B) H&E staining of normal (A) and cancerous pancreatic tissue (B) from tree shrew. is, islet; black arrow, normal duct; red arrows, glandular structures; asterisks, fibrous stroma. (C,D) Active cellular proliferation, as shown by Ki67 immunoreactivity (red). (E-L) Immunohistochemical detection of human pancreatic cancer markers CK19 (E,F), Muc5 (G,H), MMP7 (I,J) and Hes1 (K,L) (red). (M,N) Mucin proteins were stained by Alcian Blue. Normal tissue, C,E,G,I,K,M; tumor tissue, D,F,H,J,L,N.

The histological characteristics of tumor samples were examined by Hematoxylin and Eosin (H&E) staining. The tumors were moderately differentiated ductal adenocarcinoma, as evidenced by a mixture of medium-sized duct-like and tubular structures of variable shape embedded in desmoplastic stroma (Fig. 3A,B).

Cells in the tree shrew tumor mass were highly proliferative, as evidenced by extensive Ki67 expression, while only a few cells were Ki67 positive in normal tissue (Fig. 3C,D). Moreover, the tumors expressed some well-established markers of human pancreatic cancer, including the ductal markers CK19 (Fig. 3E,F), mucin 5 (Muc5) (Fig. 3G,H), matrix metalloproteinase 7 (MMP7) (Fig. 3I,J), and hairy and enhancer of split 1 (Hes1), the indicator of the active Notch signaling pathway (Fig. 3K,L). Strong Alcian Blue staining was also observed in tumors but not in normal tissue (Fig. 3M,N), revealing an abundance of mucins in the tumor. These results demonstrate that the tree shrew pancreatic cancer model recapitulates the histological and molecular features of human PDAC.

### Acinar-to-ductal metaplasia (ADM) at an early stage of pancreatic cancer in tree shrew

**Fig. 4.**
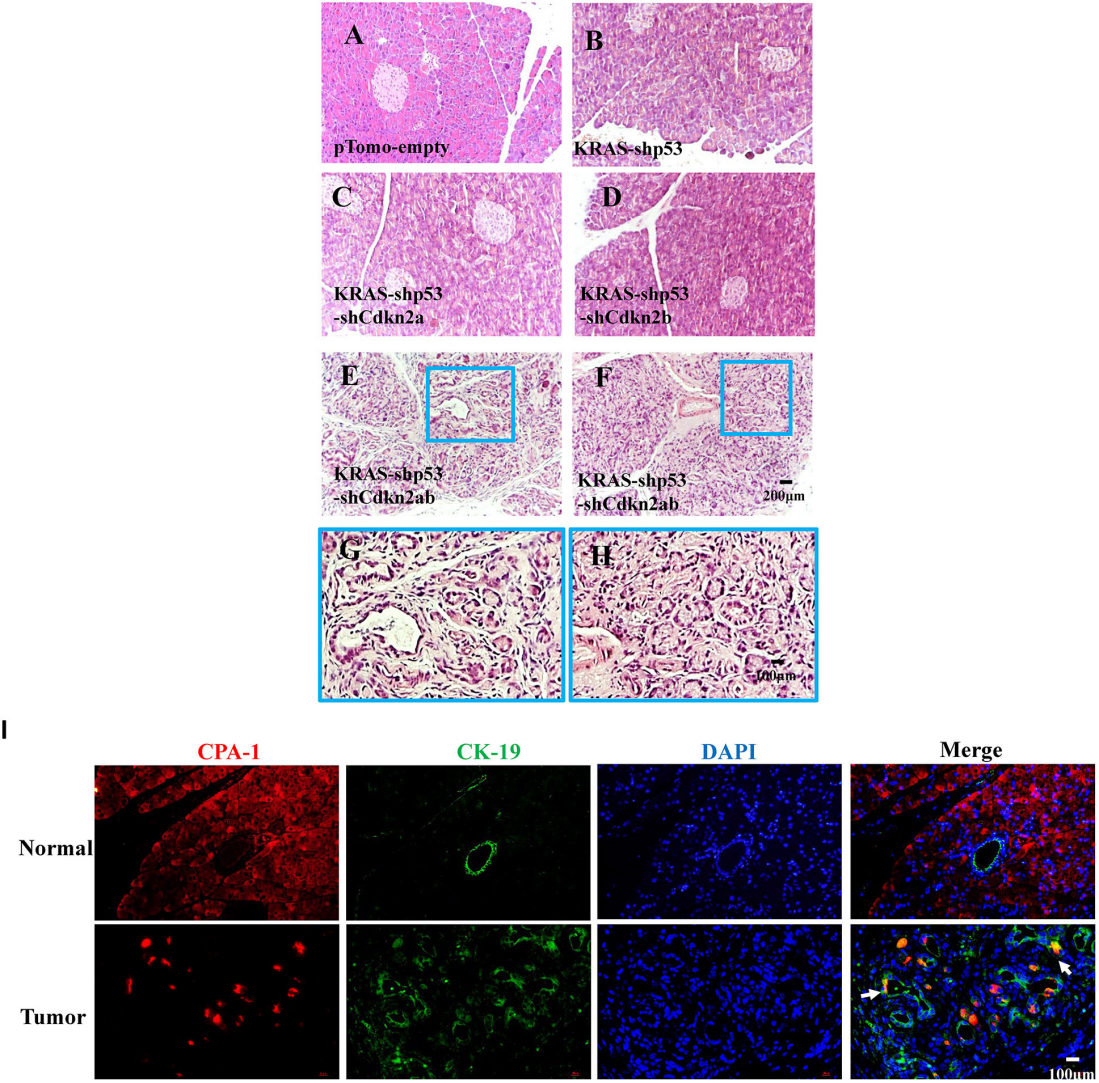
**Detection of ADM in early stages of pancreatic cancer development in tree shrew.** (A-H) Tissue was harvested 7 days after injection of the lentivirus, and H&E staining was performed in the pTomo-empty (A), KRAS-shTp53 (B), KRAS-shTp53-shCdkn2a (C), KRAS-shTp53-shCdkn2b (D) and KRAS-shTp53-shCdkn2a/b (E-H) groups. H&E staining shows an increase in the number of ducts in tissue infected by lentivirus KRAS-shTp53-shCdkn2a/b; the areas in the blue line boxes are shown enlarged below. (I) Immunohistochemical analysis of CPA1 (red) and CK19 (green) expression in normal tissue and tissue infected by lentivirus KRAS-shTp53-shCdkn2a/b; arrows indicate double-positive cells.

ADM is considered as a critical step in pancreatic cancer derived from transformation of acinar cells, and provides evidence for acinar cells as the cell of origin (Schmid, 2002). We therefore investigated whether ADM occurred at an early stage in our pancreatic cancer model. After 7 days of injection of lentivirus, the number of ducts in the pancreatic lobules increased obviously in tissues infected by KRAS-shTp53-shCdkn2a/b lentivirus, but this increase was not detected in tissues infected by other lentivirus and normal tissues (Fig. 4A-H). To observe the ADM process, double staining for CK19 and CPA1 was performed. Cells double positive for CK19 and CPA1 were prevalent in the KRAS-shTp53-shCdkn2a/b group but not in the normal group, indicating ADM progression (Fig. 4I). These results suggest that pancreatic cancer in tree shrew arises from the malignant transformation of acinar cells.

### The Rb1 signaling pathway is involved in tree shrew pancreatic cancer tumorigenesis

**Fig. 5.**
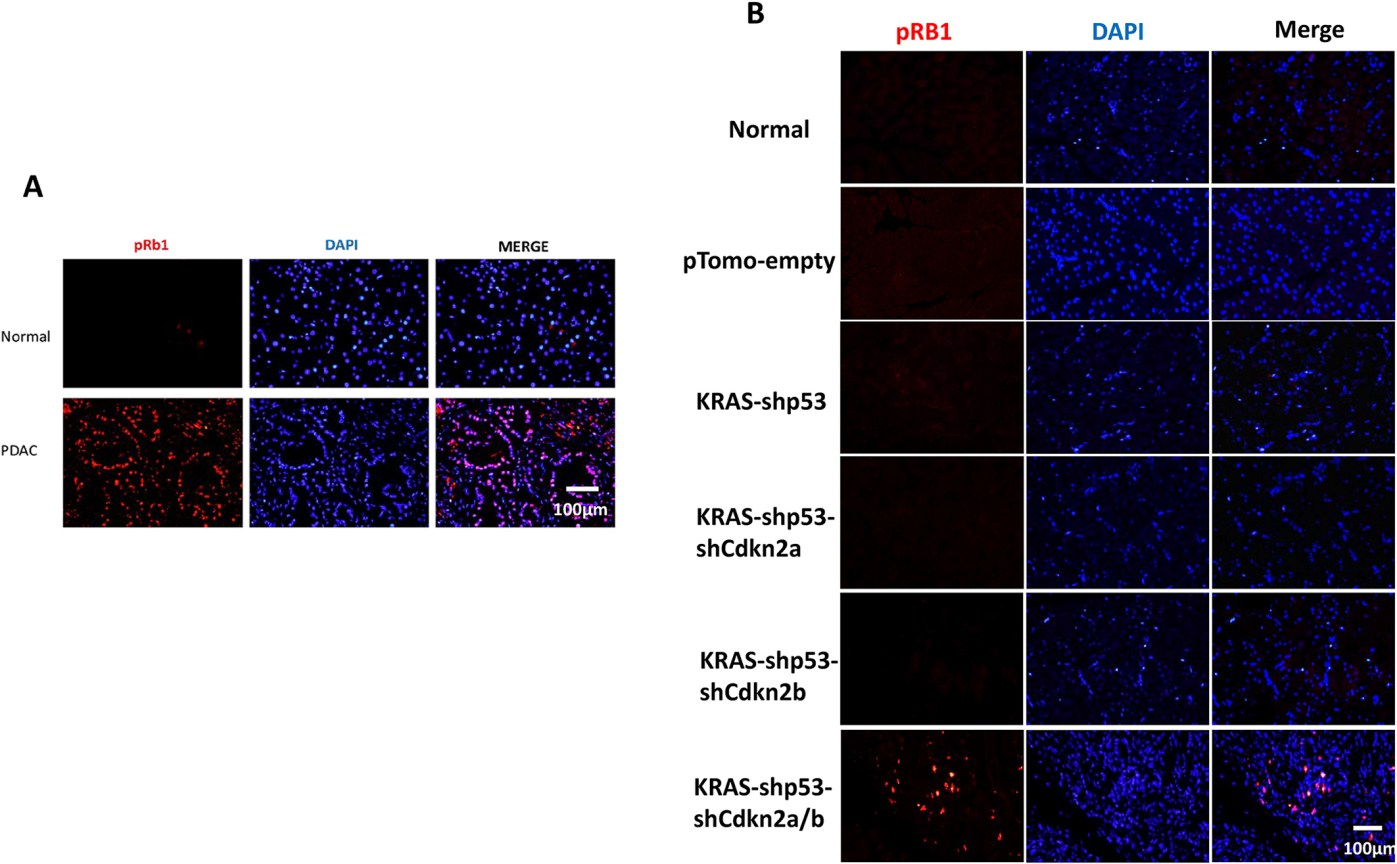
**The Rb1 signaling pathway is involved in tree shrew pancreatic cancer.** (A) Phosphorylation of Rb1 (Ser780) was detected by immunohistochemical staining in tree shrew pancreatic cancer tissue and normal tissue. (B) Phosphorylation of Rb1 (Ser780) was detected at the early stage of tree shrew pancreatic cancer; tissues were harvested 7 days after injection of pTomo-empty, KRAS-shTp53, KRAS-shTp53-shCdkn2a, KRAS-shTp53-shCdkn2b and KRAS-shTp53-shCdkn2a/b lentivirus.

Retinoblastoma 1 (RB1) was found to be frequently phosphorylated in human PDAC (Gore et al., 2016; McCleary-Wheeler et al., 2012). Both CDKN2A (P16Ink4a) and CDKN2B (P15Ink4b) could inhibit phosphorylation of RB1 at Ser780 by abrogating activity of CDK4 and CDK6. To reveal whether the inactivation of Cdkn2b is essential for phosphorylation of Rb1, and whether the Rb1 signaling pathway is involved in our tree shrew model of pancreatic cancer, phosphorylation of Rb1 (Ser780) was detected. We found that the phosphorylation of Rb1 was significantly elevated in tumor tissue, but rarely detected in normal pancreatic tissue (Fig. 5A). To confirm that the Rb1 signaling pathway contributes to tumorigenesis, phosphorylation of Rb1 (Ser780) was evaluated at an early stage of tumor formation. We found that phosphorylation of Rb1 (Ser780) was observed in tissue infected by KRAS-shTp53-shCdkn2a/b lentivirus 7 days after injection (Fig. 5B), and that the infected tissue exhibited obvious ADM pathologically. No obvious changes in Rb1 phosphorylation were found in tissues infected by other lentiviruses. These results demonstrated that the inactivation of both *Cdkn2b* and *Cdkn2a* was required for Rb1 phosphorylation, which was involved in the development of tree shrew pancreatic cancer in our model at an early stage.

### The tree shrew pancreatic cancer model is more similar to human PDAC than the mouse model

**Fig. 6.**
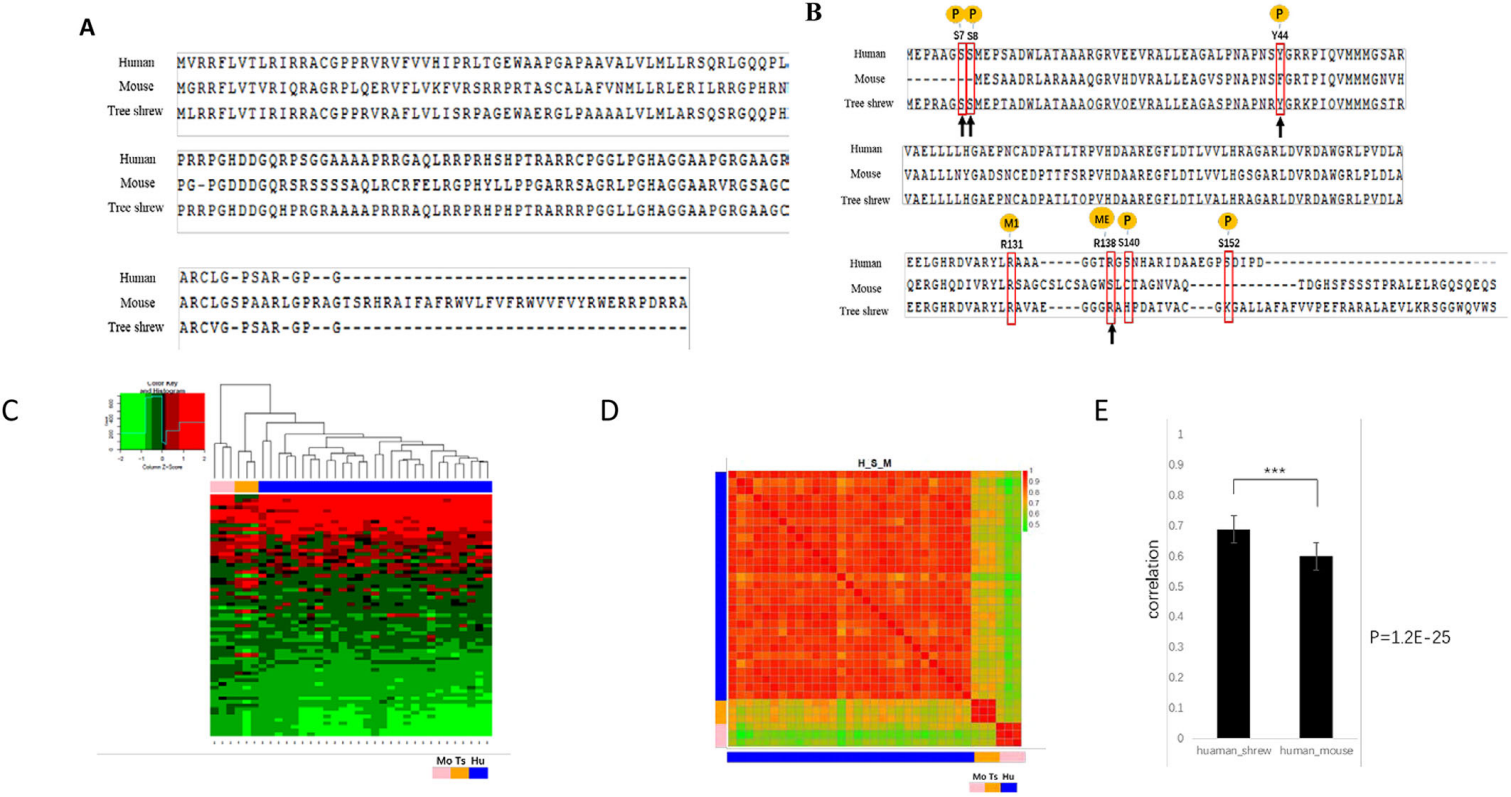
**Comparisons of human, tree shrew and mouse pancreatic cancer models.** (A) Homology comparison of ARF (encoded by *Cdkn2a*) among human, mouse and tree shrew. The homology of tree shrew to human was significantly higher than that of mouse to human. (B) Post-translational modification sites of P16Ink4a are outlined with red boxes, and the conserved sites in tree shrew, but not in mouse, are indicated by black arrows. ME, methylation; M1, mono-methylation; P, phosphorylation. (C) Pairwise comparisons of gene expression profiles of tree shrew, human and mouse PDAC. Dendrogram showing distinct clusters for the tree shrew, human and mouse samples. (D) Heat map illustrating the correlation between expression profiles of the three species. Hu, human; Mo, mouse; Ts, tree shrew. (E) Bar graph illustrating the average correlation among the three species; error bars represent s.d. ***P=1.25×10^–25^.

The tree shrew is phylogenetically more close to human than mouse. Among the genes chosen for cancer induction in this study, the protein homology and post-translational modification sites of Tp53 were analyzed previously and showed obviously higher conservation (Tong et al., 2017). The homology of *Cdkn2a*-encoded P14Arf between human and tree shrew is 81%, which is much higher than that between human and mouse (49%) (Fig. 6A). The homology of P16Ink4, another protein encoded by *Cdkn2a*, is 10% higher in tree shrew than in mouse (84% versus 74%) (Fig. 6B). We also analyzed post-translational modifications of these proteins using PhosphoSitePlus (https://www.phosphosite.org/homeAction.action) (Table S2). The S7, S8 and Y44 phosphorylation sites and R138 methylation site of P16Ink4a are conserved in tree shrew but not in mouse (Fig. 6B). Phosphorylation at the S7 and S8 of P16Ink4a was proven to activate CDK4 and promote tumorigenesis (Gump et al., 2003). Many other proteins in the Kyoto Encyclopedia of Genes and Genomes (KEGG) pancreatic cancer pathway also show higher homology to human in tree shrew than in mouse (Table S2).

To investigate gene expression in the tree shrew pancreatic cancer model, tumor samples (*n*=3) were analyzed by RNA sequencing (RNA-seq) (Fig. S1), and the expression profiles were compared with the human pancreatic cancer samples containing *KRAS*, *TP53* and *CDKN2A/B* mutations (*n*=30, from The Cancer Genome Atlas) and mouse pancreatic cancer, which was described in our previous study (Tu et al., 2017). We detected 11,541 orthologous genes across human, mouse and tree shrew in these samples (Table S3). The raw data were log-transformed and normalized using relative values (Z-scores) (Qian et al., 2010). The heatmap.2 program in R package gplots was used to evaluate differences in expression patterns among the three species, and dendrograms were constructed using the Pearson method. The global expression patterns showed three distinct clusters for tree shrew, human and mouse. Overall, expression patterns in the tree shrew model were more closely clustered with those in humans than mouse (Fig. 6C). Square matrix heat maps and pairwise bar analyses provided additional evidence that the tree shrew PDAC transcriptome is closer than that of mouse to the human counterpart (Fig. 6D,E). These results suggest that tree shrew pancreatic cancer could be more similar to the human disease in terms of molecular mechanisms than mouse pancreatic cancer.

## DISCUSSION

Both ductal and acinar cells have been proposed as the cell of origin of pancreatic cancer (Oldfield et al., 2017; Pour et al., 2003), with acinar cells, in particular, obtaining increased experimental support (Kopp et al., 2012; Maresch et al., 2016). However, the genetic alterations driving the transformation of acinar cells in adult animals remain to be deciphered, and an adult pancreatic cancer animal model originating from acinar cells has yet to be established. In this study, we found that lentivirus mainly infected pancreatic acinar cells in tree shrew and successfully established a pancreatic cancer model via malignant transformation of adult acinar cells.

Our work demonstrated that expression of oncogenic KRAS and loss of function of Tp53 and Cdkn2a/b are required for PDAC originating from mature acinar cells. Loss of function of Tp53 plus oncogenic KRAS could not induce PDAC, which was also demonstrated in work by Guerra et al. (2011, 2007), in which Tp53^lox/lox^;Elas-tTA/tetO-Cre along with expression of mutant KRAS failed to induce PDAC in the adult. Meanwhile, Bailey et al. (2016) demonstrated that the expression of KRAS^G12D^ and the gain-of-function mutant Tp53^R172H^ in adult acinar cells could induce PDAC. These results suggested that gain-of-function mutation and loss of function of Tp53 have various roles in promoting adult acinar cell transformation driven by oncogenic KRAS. Our work also demonstrated that inactivation of *Cdkn2b* was indispensable for PDAC induction, which is similar to pancreatic cancer derived from ductal cells in mice (Tu et al., 2017). The results imply that both pancreatic ductal and acinar cells are susceptible to malignant transformation by a common set of genetic alterations.

The tree shrew is a proposed experimental animal that possesses closer genetic relationship to primates than rodents. At present, there are many attempts to employ tree shrew to model a variety of human diseases. Tree shrew cancer models may have advantages over mouse models for several reasons. First, tree shrews share a close phylogenetic relationship with primates and have higher similarity to humans than do rodents at a whole-genomic level (Fan et al., 2013). Second, the majority of spontaneous tumors in tree shrew are thought to originate from epithelial cells, which is also the case in human disease (Brack, 1998), whereas many mouse strains tend to develop tumors derived from mesenchymal tissues (Rangarajan and Weinberg, 2003). Third, many cancer-associated genes are more conserved between human and tree shrew. As shown in this study, several genes in KEGG pancreatic cancer pathways are more similar to their human homologs in terms of protein sequence and post-translational modification sites, including Tp53 (Tong et al., 2017), P16Ink4a/P14Arf and P15Ink4b, which are most frequently mutated in human PDAC and were applied to induce pancreatic cancer in this study. Finally, the expression profile of the tree shrew PDAC model was more similar than that of mouse to human samples, as for the glioblastoma model in our previous study (Tong et al., 2017).

In summary, we established a new pancreatic cancer model in tree shrew by malignant transformation of acinar cells via infection with a lentivirus expressing the mutant KRAS^G12D^ and loss of function of *Tp53* and *Cdkn2a/b* by shRNAs. This model recapitulates the major histological features and expresses the molecular makers of human PDAC. It can provide a model of adult pancreatic cancer by recapitulating genetic mutations in human disease, and could potentially be of benefit in basic pancreatic cancer research and anti-tumor drug discovery.

## MATERIALS AND METHODS

### Tree shrews

Adult male Chinese tree shrews (*Tupaia belangeri chinensis*; 2-3 years old, weighing 120-150 g) were obtained from the Kunming Primate Research Center, Kunming Institute of Zoology, Chinese Academy of Sciences. Animal care and experimental protocols were approved by the Animal Care and Use Committee of Kunming Institute of Zoology.

### Construction of lentiviral vectors

The lentiviral vectors used in this study contained the mutant oncogene KRAS^G12D^ and shRNAs targeting *Tp53*, *Cdkn2a*, *Cdkn2b* and *Cdkn2a/b*. These elements were inserted into the pTomo vector (Addgene, Cambridge, MA, USA; cat. no. 26291) as per the following description. The red fluorescent protein-IRES-enhanced green fluorescent protein fragment (RFP-IRES-EGFP) was replaced with the *KRAS^G12D^* gene by *Xba*I and *Sal*I digestion. shTp53 was inserted into the *Cla*I restriction site upstream of the cytomegalovirus (CMV) promoter to generate a vector KRAS-shTp53. shRNAs targeting *Cdkn2a*, *Cdkn2b* or *Cdkn2a/b* were then inserted downstream of KRAS^G12D^ between the *Age*I and *Sal*I restriction sites to obtain the KRAS-shTp53-shCdkn2a, KRAS-shTp53-shCdkn2b and KRAS-shTp53-shCdkn2a/b vectors. The shRNA target sequences were CACCATCCACTACAACTACAT for *Tp53*, GGCTTCTTGGACACGCTGGTG for *Cdkn2a* and GCAGATCCCAACGGAGTCAAC for *Cdkn2b*. CCCGGGAGGGCTTCTTGGACAC was used as a target for both *Cdkn2a* and *Cdkn2b*. An shRNA targeting the luciferase gene in the pTomo-KRAS-shLuc vector served as the shRNA control.

### Lentiviral particle preparation

Lentiviral particles were packaged as described previously (Tu et al., 2017). The pCMVΔ8.9 and pMD2.G vectors were introduced into HEK293T cells at a ratio of 5:2.5:1 by polyethylenimine-mediated transfection. The medium was replaced with fresh medium 6 h after transfection, harvested at 48 h and 72 h, and centrifuged at 3000 ***g*** for 10 min to remove cellular debris, followed by centrifugation at 80,000 ***g*** at 4°C for 2.5 h. The pellet containing viral particles was dissolved in phosphate-buffered saline containing 0.1% bovine serum albumin. Aliquots of the lentivirus were stored at −80°C.

### Lentiviral injection

Tree shrews were anesthetized with ketamine at a dose of 100 mg/kg. The abdominal hair was removed with clippers and the skin surface was sterilized with iodine. A 1-cm incision was made and the head of the pancreas was injected with 20 μl lentivirus containing 0.1% Trypan Blue using a 30-gauge needle. The incision was sutured, and animals were maintained under a heating lamp until recovery from anesthesia.

### Histopathological analysis and immunohistochemistry

Fresh pancreatic tissue specimens were dissected and immediately fixed in 4% formalin for 48 h or rapidly frozen in liquid nitrogen. Paraffin-embedded sections (4 μm) were stained with H&E for histopathological analysis. Immunohistochemistry was performed as previously described (Yang et al., 2017). Primary antibodies against the following proteins were used in this study: CK19 (Proteintech, Rosemont, IL, USA; cat. no. 10712-1-AP) and CPA1 (Proteintech; cat. no. 15836-1-AP); Ki67 (Vector Laboratories, Burlingame, CA, USA; cat. no. VP-K452); Muc5 (Novacastra, Newcastle-upon-Tyne, UK; cat. no. NCL-MUC-5AC); Hes1 (Aviva Systems Biology, San Diego, CA, USA; cat. no. ARP32372_T100); MMP7 (Vanderbilt Antibody and Protein Resource, Nashville, TN, USA) (Crawford et al., 2002); Rb-phosphor S780 (Abcam, Cambridge, UK; cat. no. ab47763); and EGFP (Abcam; cat. no. ab13970). Fluorophore-conjugated secondary antibodies were purchased from Life Technologies (Carlsbad, CA, USA).

### Western blotting

Cells were lysed with RIPA buffer and total protein was quantified with a BCA kit (Beyotime Institute of Biotechnology, Shanghai, China; cat. no. P0009). Proteins were separated by SDS-PAGE followed by immunoblotting with primary antibodies against KRAS^G12D^ (NewEast Biosciences, Malvern, PA, USA; cat. no. 26036); P15 (cat. no. 12877-1-AP) and P16 (cat. no. 10883-1-AP) (both from Proteintech); and P53 (Santa Cruz Biotechnology, Santa Cruz, CA, USA; cat. no. sc-6243). Specific signals were detected with horseradish-peroxidase (HRP)-conjugated secondary antibodies (Sigma-Aldrich, St. Louis, MO, USA) and chemiluminescent HRP substrate reagent (Millipore, Billerica, MA, USA; cat. no. WbKLS0500).

### Quantitative real-time (qRT-)PCR

Total RNA was isolated with TRIzol reagent (Sigma-Aldrich). Reverse transcription was carried out using a RevertAid First Strand cDNA Synthesis Kit (Thermo Fisher Scientific, Waltham, MA, USA), and qRT-PCR was performed with an SYBR Select Master Mix Kit (Life Technologies).

### RNA-seq

Total RNA was extracted from three tree shrew pancreatic tumor samples (#130, #149 and #153). RNA-seq libraries were constructed using the mRNA-Seq Prep kit (Illumina, San Diego, CA, USA) and sequenced on the Illumina HiSeq 4000 platform in paired-end form with 150 bp (Macrogene, Seoul, South Korea). Approximately 7 Gb of raw, high-quality sequence data were obtained for each sample (Table S1). Raw data and gene expression data have been deposited in the National Center for Biotechnology Information (NCBI) Gene Expression Omnibus (GEO) (accession no. GSE106343). Bowtie software was used for sequence alignment, and the upper-quartile normalization method was used to calculate gene expression values for each sample. Hierarchical clustering of samples was performed with R package (v.3.2.2) gplots (https://www.R-project.org/).

## Supplementary Material

Supplementary information
