# Supplementary material for: A novel pancreatic cancer model originated from transformation of acinar cells in adult tree shrew, a primate-like animal
Source: Dis Model Mech. 2019 Apr 15;12(4):dmm038703. doi: 10.1242/dmm.038703 (PMC6505477; doi:10.1242/dmm.038703)
Supplement: Supplementary information [file dmm-12-038703-s1.pdf]

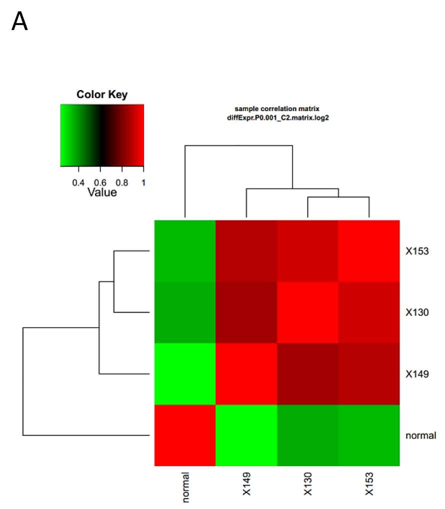

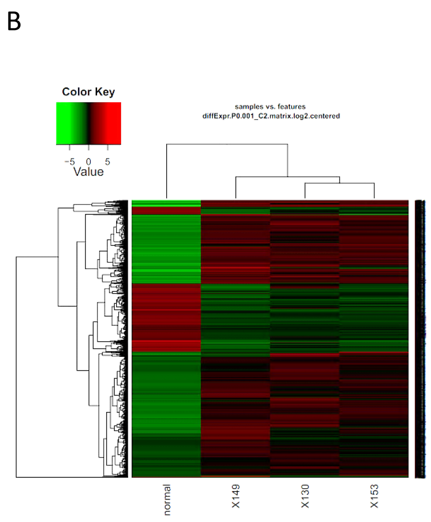

Figure S1. Analysis of RNA-seq of tree shrew pancreatic cancer. (A) sample correlation of three tumor samples and normal tissue. (B) differentially expressed genes between tumor samples and normal tissue.

Disease Models & Mechanisms • Supplementary information

Table S1. The quality of RNA-seq of tree shrew samples.

Click here to Download Table S1

Table S2. Homology between human, tree shrew, and mouse pancreatic cancer pathway genes and their modification sites.

Click here to Download Table S2

Table S3. The relative expression of human-Tree shrew-mouse 1:1:1 ortholog genes.

Click here to Download Table S3
